# A Compact Multiphoton 3D Imaging System for Recording Fast Neuronal Activity

**DOI:** 10.1371/journal.pone.0000699

**Published:** 2007-08-08

**Authors:** Dejan Vučinić, Terrence J. Sejnowski

**Affiliations:** 1 Howard Hughes Medical Institute, Computational Neurobiology Laboratory, The Salk Institute for Biological Studies, La Jolla, California, United States of America; 2 Division of Biological Sciences, University of California at San Diego, La Jolla, California, United States America; National Institutes of Health, United States of America

## Abstract

We constructed a simple and compact imaging system designed specifically for the recording of fast neuronal activity in a 3D volume. The system uses an Yb:KYW femtosecond laser we designed for use with acousto-optic deflection. An integrated two-axis acousto-optic deflector, driven by digitally synthesized signals, can target locations in three dimensions. Data acquisition and the control of scanning are performed by a LeCroy digital oscilloscope. The total cost of construction was one order of magnitude lower than that of a typical Ti:sapphire system. The entire imaging apparatus, including the laser, fits comfortably onto a small rig for electrophysiology. Despite the low cost and simplicity, the convergence of several new technologies allowed us to achieve the following capabilities: *i*) full-frame acquisition at video rates suitable for patch clamping; *ii*) random access in under ten microseconds with dwelling ability in the nominal focal plane; *iii*) three-dimensional random access with the ability to perform fast volume sweeps at kilohertz rates; and *iv*) fluorescence lifetime imaging. We demonstrate the ability to record action potentials with high temporal resolution using intracellularly loaded potentiometric dye di-2-ANEPEQ. Our design proffers easy integration with electrophysiology and promises a more widespread adoption of functional two-photon imaging as a tool for the study of neuronal activity. The software and firmware we developed is available for download at http://neurospy.org/ under an open source license.

## Introduction

Two-photon imaging [1] is a powerful technique for recording the activity of large populations of neurons from deep within living tissue [2], [3]. The promise of simultaneously following large numbers of individual cells identified through transgenic labels [4], as well as the ability to see neurons' internal states beyond action potential firing [5], makes this optical approach ideal for study of network dynamics and plasticity. Moreover, the ability of multiphoton excitation to yield sharp images from deep within scattering tissue enables the study of microscopic processes *in situ* [6]–[8].

Nonlinear imaging of neuronal activity also raises great challenges. To observe changes at millisecond timescales over durations long enough to be physiologically relevant requires a careful selection of excitation intensities and detection techniques so that the fluorescence photon yield, penetration depth, heating and photodamage are all optimized. This is a fundamental limitation of the very small, femtoliter-sized, volume where multiphoton absorption is induced. For instance, there are only 602 molecules of a fluorescent reporter at 1 µM in a femtoliter, so if these are excited at photodamage-limited intensities [9], [10] there are very few photons per unit time emerging from the focal spot, making shot noise a severe limitation on the ability to observe small, fast changes.

One particularly daunting problem has been the recording of neuronal membrane potentials using voltage-sensitive dyes [11]. Because of the shot noise limitations, labels that undergo relatively larger changes in response to membrane potential fluctuations, such as second-harmonic upconversion with FM 4-64 [12], have a significant advantage over the more widely used styril dyes [13]–[17]. These techniques, however, are not useful for voltage imaging *in vivo*, and still incur a rapid deterioration of tissue resulting from very high intensities of excitation light required. New fluorescent dyes have been developed [18] that give much larger voltage-sensing signals [19], but their use has been hindered by the difficulty of delivery into tissue and the very limited availability of lasers suitable for excitation at wavelengths where their sensitivity to transmembrane voltage is high.

In this article we describe an apparatus we constructed that can record smaller, faster changes of fluorescence signals. We designed the entire imaging system for this single purpose, ignoring microscopy traditions and so eliminating a number of practical constraints that would have prevented us from reaching the physical limits of materials and the technologies available. The Ytterbium laser we constructed operates in a longer-wavelength region where many functional fluorescent reporters have high sensitivities and where light penetrates tissue deeper. We selected longer laser pulses for compatibility with very fast but highly dispersive acousto-optic deflectors, and as a result ended up with lower photodamage. We exploited the latest developments in Direct Digital Synthesis of radio-frequency signals to achieve fast three-dimensional scanning with no moving parts, and to reach the physical limits of acousto-optic materials in their ability to reposition the beam and control its intensity.

Finally, we exploited the amazing increase in the computing power and data acquisition bandwidth of modern oscilloscopes to eliminate the need for a dedicated microscope controller. This resulted in several unanticipated benefits: the cost of construction of the entire imaging system is reduced by an order of magnitude compared with today's prevailing designs, down to approximately forty thousand 2006 U.S. dollars in parts including the laser and vibration isolation; the daily operational complexity is greatly reduced compared with a typical imaging system integrated from disparate components, facilitating dedicated deployment; and the data acquisition system is fast enough for fluorescence lifetime imaging, even outperforming many dedicated solutions at a fraction of the cost.

## Results

**Figure 1:**
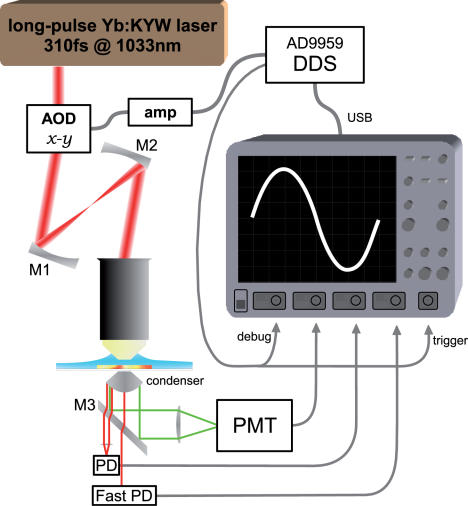
The diagram of our simple imaging system. A LeCroy WaveRunner 64xi oscilloscope acts as both the scan controller and the data acquisition system. Radio-frequency signals generated by the AD9959 Direct Digital Synthesis chip, which is controlled by the oscilloscope via USB, are amplified and injected into a compact two-axis acousto-optic deflector. Long laser pulses have narrow spectral bandwidth and so obviate the need for dispersion compensation. The use of mirrors M1 and M2 instead of lenses allows for a very compact microscope. Mirror M3 steers fluorescence collected by the condenser to the photomultiplier (PMT). A fast photodiode (PD) collects transmitted excitation light that was deflected to large angles to provide oblique contrast for observing cells in non-fluorescent tissue. A very fast photodiode (FastPD) reports laser pulse timing for fluorescence lifetime measurements.

**Figure 2:**
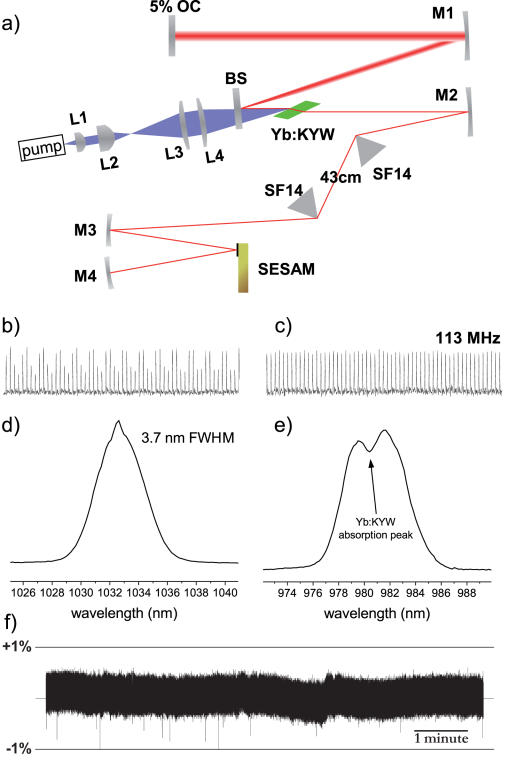
Design and performance of the Yb:KYW laser. a) Diagram of the laser cavity. Pump light is collimated and focused by lenses L1–4 to a 51 µm × 85 µm waist in air; pump beam is tilted relative to the lasing beam to keep reflections from reentering the pump diode and destabilizing it. Beamsplitter (BS) has high transmissivity at 981 nm and high reflectivity at >1010 nm. Lasing medium is a d = 1.2 mm Brewster-cut 10%-at. Yb:KYW crystal. M1,M4 = −200 mm, M2,M3 = −100 mm are cavity mirrors with standard λ/4 dielectric coating. SF14 – uncoated isosceles prisms. OC – output coupler. b) Low pump beam quality makes the laser prone to multimode operation, which is easily observed by a photodiode as the circulation of pulse energy between lobes of higher-order modes on subsequent passes. c) Restricting the cavity to single-mode operation results in even pulsing at 113 MHz with 300 mW of average power. d) Output spectrum is 3.7 nm wide and centered near 1033 nm, indicating sech^2^ transform-limited pulse width of 310 fs. e) Spectrum of the transmitted pump light. The narrow Yb:KYW absorption peak near 981 nm is readily visible; the pump wavelength must be temperature-tuned to overlap it for maximum output power and stability. f) Output power variability. With the off-axis pumping arrangement the laser is capable of very quiet operation.

Figure 1 shows a diagram of our imaging apparatus. Excitation light was generated by a femtosecond laser (Figure 2) that used Ytterbium-doped KY(WO_4_)_2_ (Yb:KYW) as the lasing medium. The beam was deflected for scanning by an integrated two-dimensional acousto-optic deflector (Brimrose 2DS-50-30-1.06, www.brimrose.com), and mapped onto the back-aperture of an objective using two concave mirrors that replace the scan lens and the tube lens in a traditional microscope. The deflector, the mirrors and the objective were mounted onto a small stage moved by a Sutter MP-285 micromanipulator. There was no microscope other than this moving stage. The laser and the moving stage were mounted onto a 1 ft.×2 ft. breadboard (Thorlabs PBH11102) which was elevated above the working surface to allow placement of the specimen holder and the detection equipment underneath the objective. The working surface was suspended on a negative-stiffness passive vibration isolation platform (MinusK Technology 250BA-1, www.minusk.com). The entire apparatus, including vibration isolation and the laser, measured 3 ft. wide by 2.5 ft. deep by 3 ft. high.

**Figure 3:**
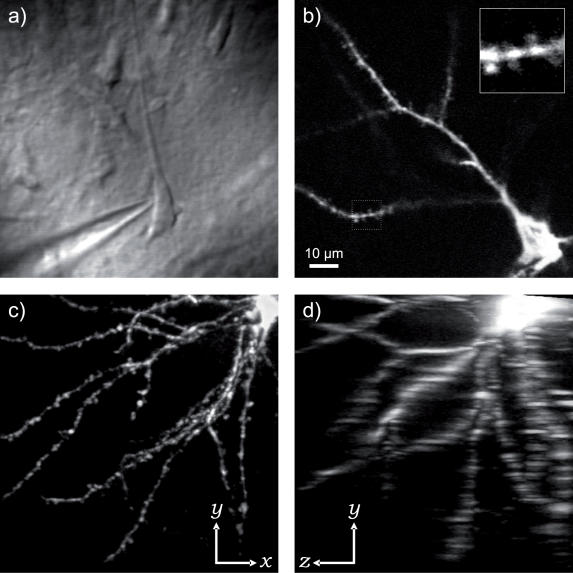
Imaging performance and resolution. a) Transmitted light image of a neuron in acute rat brain slice. Video-rate acquisition permits patch-clamping without the use of a separate camera. b) Fluorescence image of a cortical L2/3 pyramidal cell filled with the fluorescent dye FM 4–64. Inset: zoom onto the 10×10 µm area outlined in the image showing dendritic spines. c,d) *x*–*y* and *z*–*y* projections of a 100 µm deep stack of one quadrant of the basal dendritic tree of a cortical L2/3 pyramidal cell filled with the voltage-sensitive dye di-2-ANEPEQ. Thin processes at 100 µm depth can be observed easily. The axial resolution is worse than the objective's limit because we deliberately underilluminate the back aperture. All images were acquired with a 60×0.9NA water-immersion objective and have 100×100 µm field of view.

For thin preparation work, transmitted fluorescence was collected by a condenser and steered using a broadband dielectric mirror (Newport 13E20BD.1) to a photomultiplier module (Hamamatsu 7422-40). Excitation light passed through this mirror and a portion deflected to large angles was collected by a fast photodiode (35 MHz with preamplifier, Hamamatsu S6468-02), which provided enough contrast to see cells in non-fluorescent tissue (Figure 3a). The condenser could be mounted onto the *x-y* portion of the microscope manipulator, but we left it stationary for simplicity since it allowed access to a region 2 mm wide without having to move the specimen.

For imaging of epifluorescence *in vivo* or in thick preparations a dichroic mirror could be placed at the back-aperture of the objective to steer epifluorescence onto an appropriate detector, such as an avalanche photodiode, mounted close to the objective to maximize the collection efficiency. For this type of experiment everything below the objective could be replaced by a stereotaxic device, and the folded microscope tube after the deflector could be tilted to permit access to tissue at an angle.

### Construction of an Yb:KYW femtosecond laser

Figure 2a shows a diagram of the Yb:KYW laser. Pump power was provided by a single InGaAs/AlGaAs laser diode (Axcel Photonics CM-980-3500-150, www.axcelphotonics.com) with a 100 µm wide emitter producing 3.5 W of multimode output with a 5 nm wide spectrum temperature-tuned to 981 nm (Figure 2e), the absorption peak of Yb:KYW. The diode was powered by a generic lab power supply (Instek PSP-2010). Diverging pump light was collimated by an aspheric pick-up lens (Geltech 350350, f = 4.5 mm, 0.41 NA, www.thorlabs.com) and the slow axis magnified by two cylindrical lenses (Thorlabs LJ1805L1-B and LJ1934L1-B) to achieve tighter focus. The pump beam was focused onto the lasing medium with an f = 60 mm spherical singlet lens (Thorlabs LA1134-B) through a beamsplitter with a coating designed for high transmissivity at 981 nm and high reflectivity at >1010 nm (EKSPLA, Vilnius, Lithuania, www.ekspla.com). The pump beam waist measured 51 µm (fast axis 1/e^2^ Gaussian) by 85 µm (slow axis FWHM flat-top) wide in air. Careful shaping of the pump beam was essential for achieving the low lasing threshold and high output power.

The lasing medium was a Brewster-cut Yb:KYW crystal (4×4 mm, d = 1.2 mm, 10%-at., Altechna, Vilnius, Lithuania, www.altechna.lt). Laser cavity mirrors had standard λ/4 dielectric coatings centered near 1064 nm (CASIX, Inc., Fuzhou, China, www.casix.com); the output coupler (EKSPLA) had 5% transmissivity at 1030 nm. For modelocking we used a SESAM^27,28^ with 0.4% modulation depth at 1040 nm, 120 µJ/cm^2^ saturation fluence and 1 GW/cm^2^ damage threshold (SAM-1040-0.7-25.4g, BATOP GmbH, Weimar, Germany, www.batop.de). For the correction of group velocity dispersion we used isosceles SF14 prisms (CASIX, Inc.); tip-to-tip prism separation was 43 cm.

The maximal continuous-wave multimode output power we measured with this pumping arrangement was 1025 mW near 1037 nm, achieved by using a 6% output coupler, no SESAM or prisms in the cavity, on-axis pumping, running the diode at higher than nominal power (3.8 W), and by cooling the crystal below room temperature. In the final modelocked configuration used for imaging the pump beam was tilted off-axis to keep reflected pump light from reentering the diode, which would destabilize it; off-axis pumping allowed very quiet operation (Figure 2f) at the expense of some extraction efficiency. More power was lost through reflections at prism surfaces and at the SESAM which, in addition to the 0.4% modulation depth, had 0.3% non-saturable losses. Two passes on the SESAM and a 5% output coupler yielded 300 mW of quiet single-mode femtosecond output at 113 MHz repetition rate (Figure 2c). The spectrum was centered near 1033 nm and 3.7 nm wide (Figure 2d), indicating transform-limited sech^2^ pulse width of 310 fs. Such long pulses obviated the need for compensation of spatial and temporal dispersion when using an acousto-optic deflector for scanning.

Detailed instructions for the construction of our laser are available from the braintool wiki at http://braintool.org/optical/spy1/.

### Acousto-optic scanning with Direct Digital Synthesis

We used a standard two-axis acousto-optic deflector (AOD) made by Brimrose Corporation (2DS-50-30-1.06, www.brimrose.com). This deflector uses two TeO_2_ crystals in slow-shear mode (620 m/s speed of sound), has a nominal aperture of 7×7 mm, and comes in a single housing conveniently pre-aligned for collinear input beam and first-order diffracted output beam, which greatly simplified the design and alignment of the microscope. The displacement in space (∼30 mm) of the crystals for *x* and *y* deflection implies a small (<10%) beam runoff at the second crystal at deflection limits, but we under-illuminated the aperture with a ∼4 mm wide beam resulting in effectively no power loss from run-off.

Radio-frequency signals required to drive the AOD were produced by a Direct Digital Synthesis (DDS) chip (AD9959, Analog Devices, www.analog.com). This device provided four output channels that could independently generate frequencies between 0 and 250 MHz with 32-bit resolution, with 10-bit amplitude and 14-bit phase resolution. Two of the output channels were used to drive the AOD, one to trigger data acquisition, and the remaining channel was used to debug scanning protocols. The deflecting signals were amplified by RF amplifiers (Mini-Circuits ZHL-1-2W-S, www.mini-circuits.com) which produced 1 W of output power, resulting in the total diffraction efficiency of approximately 21%, one half of the nominal 42%.

**Figure 4:**
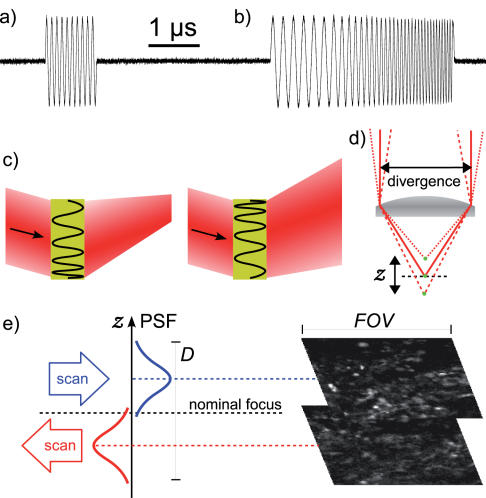
Fast digital control of acousto-optic deflection enables three-dimensional scanning with no moving parts. Direct Digital Synthesis permits a) precise control of radio-frequency signals at microsecond timescales, as well as b) accurate and exactly repeatable sweeping between preset frequencies. The nominal aperture of the deflector we use is approximately the size of the entire trace in a,b). c) A frequency sweep makes an acousto-optic deflector act as a lens, so the output beam can be made to converge or diverge depending on the direction of the sweep. d) The lensing induced in this manner translates into axial displacement of the focus from the nominal focal plane of the objective. e) Large volumes can be scanned rapidly through the use of bidirectional line sweeps. By underilluminating the back-aperture of an objective the axial point-spread function (PSF) can be extended to match the excursion from the nominal focal plane caused by the frequency sweep, so a sweep in one direction can be made to excite a large number of sparsely labeled neurons in a thick slab of tissue to either side of the nominal focal plane. The images show two focal planes, displaced by 40 µm in *z*, of EYFP-labeled neurons in mouse olfactory bulb taken simultaneously without moving the objective.

Frequency, amplitude or phase of the generated output waves could be changed programmatically in less than one microsecond (Figure 4a). Most importantly, frequency could be swept smoothly between two presets (Figure 4b), thus allowing the injection of a modulated diffraction pattern into the AOD. This feature enabled two important scanning modes: first, a slower frequency sweep that allowed fast line-based scanning [20] of full frames at video rates; second, a faster frequency sweep was used to inject a highly modulated diffraction pattern that deflects to different angles at different parts of the AOD aperture, effectively lensing the output beam [20] and allowing departure from the nominal focal plane, i.e. 3D scanning (Figure 4c, d).

The AD9959 DDS chip we used was on an evaluation board (AD9959/PCB, www.analog.com) with USB connectivity and a microcontroller. While the control of scanning could be entirely off-loaded to the microcontroller, we found this mode to be relatively slow. We wrote custom firmware to take advantage of a much faster data-transfer mode, which allowed us to modify the waveform generated by the DDS chip in less than a microsecond (Figure 4a). The firmware and the requisite device drivers are available for download from http://neurospy.org/.

### Scan control and data acquisition with an oscilloscope

The LeCroy WaveRunner 64xi oscilloscope, running Microsoft Windows XP, was the only computer in the imaging system. It comes integrated into a single compact box with the data acquisition subsystem, knob control panel and a touch-screen display, with a variety of standard connectors (mouse, keyboard, Ethernet, USB) provided on the side. A second monitor could be connected to the oscilloscope to extend the total screen estate from the 800×600 pixels provided on the built-in display. The acquisition subsystem had a 600 MHz analog bandwidth and the maximal sampling rate of 10 GS/s (billion samples per second) on two channels or 5 GS/s on all four channels simultaneously. A total of 25 MS (million samples) could be acquired in a single sweep on two channels, or 12.5 MS on all four.

**Figure 5:**
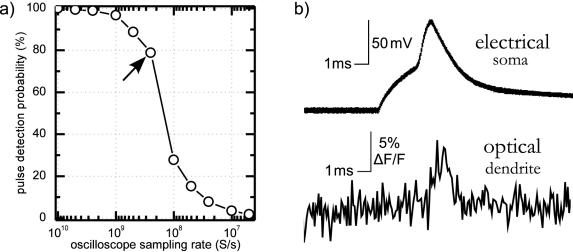
Point-dwelling ability and photon counting permit optical recording of fast events with good signal-to-noise ratio. a) The measured probability of detecting a photomultiplier pulse as a function of the oscilloscope sampling rate for a Hamamatsu 7422-40 module biased to 1000V at 2.5mV discrimination threshold. With 25 MS of sample memory and 250 MS/s sampling rate (arrow), photons can be counted for 100 ms per episode with 80% detection probability. b) Electrical and unfiltered optical (10 kHz) traces of an action potential in a rat cortical pyramidal neuron loaded with the potentiometric dye di-2-ANEPEQ. The traces are averages of four recordings.

Most detectors commonly used in microscopy could be connected directly to this oscilloscope, without additional amplification. Notably, any input could be set to 50 Ω impedance, allowing direct connection from the output of the Hamamatsu 7422-40 photomultiplier module we used for fluorescence detection. At the highest sensitivity setting (2 mV/div) the oscilloscope could readily count individual photons, which produce pulses of electrons a few nanoseconds long. For instance, the 25 MS of sample memory could be filled at 250 MS/s to count photons with 80% efficiency for 100 ms at a time (Figure 5a). Data could be acquired piecemeal in up to 10000 segments that could be triggered at arbitrary intervals, offering great flexibility in designing scanning protocols and simplifying the demultiplexing of random-access scans. Photon counting significantly improved the signal-to-noise ratio of dim fluorophores over DC current measurement, while the DC mode could be used with bright samples without any modifications to the hardware by software-switching the oscilloscope input to 1 MΩ impedance. For example, if the connecting cable was terminated with a 10 kΩ resistor the decay constant of the DC signal from the photomultiplier was on the order of a microsecond, so episodes 25 seconds long could be acquired at 1 MS/s. Such cable termination could be left in place permanently, as it did not cause a significant impedance mismatch at 50 Ω.

We wrote an application framework, neurospy, which can be run on the oscilloscope to control the scanning and data acquisition. neurospy is available for download from the project web site at http://neurospy.org/.

### Low-noise recording of fast voltage transients

The combination of point-dwelling ability, photon counting and a low-noise source of excitation light enabled two-photon recording of fast voltage transients using several styril dyes which report changes in transmembrane voltage through the change in absorption cross-section at the red spectral edge. In Figure 5b we show an example of a signal we recorded from the apical dendrite of a rat cortical pyramidal neuron in acute brain slice, filled intracellularly with the voltage-sensitive dye di-2-ANEPEQ [14] as described in ref. [16]. The neuron was patch-clamped onto the soma and held in current clamp. Single action potentials were induced by current injection synchronized with episodic optical recording. Laser scan was held at a single location on the apical dendrite throughout the recording. The shown 10 kHz optical trace is a sum of four such recordings; the raw data can be downloaded from the project web site. Photon shot noise was the dominant source of noise in the optical trace: the cumulative photon count at resting voltage was only 4500 photons per 100 µs sample. Accurate measurement of the rate of dye bleaching was precluded by systematic effects such as tissue movement, dye diffusion and slow laser noise; we conservatively estimate it to be less than 30% after one hour of continuous full-frame scanning at the maximum excitation power available to us. Such a low rate of photobleaching suggests that a significant improvement in fluorescence yield could be achieved with a more powerful laser. This method of measuring transmembrane voltage therefore holds the promise of reaching signal-to-noise ratios comparable to those achieved with quiet cameras and widefield illumination [15], [16] while benefiting from all the advantages of two-photon excitation: the ability to see much deeper into tissue than with widefield single-photon excitation (Figure 3d), as well as relative insensitivity to tissue autofluorescence and out-of-focus labeling resulting from the optical sectioning inherent in non-linear absorption. The current practical limit on the noise levels with this mode of recording was the maximal light intensity photomultipliers can sustain without damage—approximately 10^7^ photons per second.

### Fluorescence lifetime imaging

**Figure 6:**
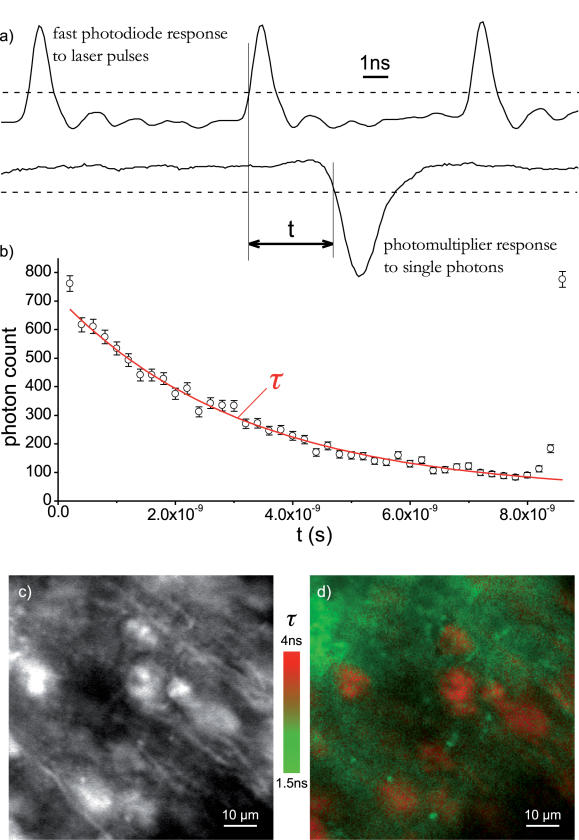
Fluorescence lifetime imaging. a) The high temporal resolution of oscilloscope data acquisition enables the measurement of temporal delay between an exciting laser pulse and a fluorescence photon. A histogram b) of relative arrival times measured in this manner can be fit to measure the fluorescence lifetime of fluorophores at the scanned location. By performing this measurement at every pixel (or voxel) a fluorescence lifetime image is constructed. c) Mouse hippocampal tissue loaded extracellularly with the dye FM 4–64. d) Fluorescence lifetime image clearly reveals Thy1.2-EYFP neurons (orange) within the background staining (green). Video-rate imaging of transmitted light or of fluorescence intensity can be used for guided patching of neurons that are first identified by their fluorescence lifetime.

The minimal sampling interval of the oscilloscope we used, 100 picoseconds, is short enough to permit accurate recording of the timing of laser pulses, which we monitored using a fast photodiode (Thorlabs D400FC). By measuring the delay between the onset of a photomultiplier pulse and the preceding laser pulse, as illustrated in Figure 6a, we were able to construct histograms of fluorescence decay latencies (Figure 6b) while scanning a given location and so to measure the fluorescence lifetime of the fluorophores present within the excited volume. By repeating this measurement at each pixel we could construct a fluorescence lifetime image, such as that shown in Figure 6d. Fluorescence lifetime imaging could be performed during 3D scanning. While the acquisition of one fluorescence lifetime image takes several minutes, video-rate imaging of fluorescence intensity could be used for guided patching of neurons identified by their fluorescence lifetime.

### Three-dimensional scanning

Fast DDS control of the radio-frequency patterns injected into the AOD enabled three-dimensional scanning without moving the objective. The amount of excursion from the nominal focal plane depended on the objective used. The effective focal length of the deflector during a frequency sweep is to first approximation given by:
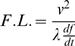
where *v* is the speed of sound in the acousto-optic material, *λ* is the wavelength of light used, and *df / dt* is the rate of frequency sweep. A full-bandwidth sweep over the nominal aperture size of our deflector resulted in the effective focal length limits of |*F*.*L*.|>140 mm.

We implemented a practically useful three-dimensional volume scan pattern, depicted in Figure 4e. By sweeping between the extremes of deflector bandwidth at a given rate *df / dt* different focal planes could be scanned rapidly. A frequency sweep in opposite directions results in displacement of the focal plane in opposite directions. By appropriately underilluminating the back aperture of an objective the point-spread function could be stretched in the *z* direction to match the excursion from the nominal focal plane caused by the frequency sweep. In this way a thick slab of tissue could be scanned rapidly. For instance, with a 40x objective a Δf = 30 MHz sweep of 25 µs duration displaces the focal plane by nearly ±30 µm, so a field of view FOV = 180 µm by D = 120 µm deep could be swept at over 100 volumes per second at the resolution limit. Similarly, smaller volumes could be swept at kilohertz rates. Again, the practical limitation of this scanning method was the amount of fluorescence that could be produced and detected during the very rapid scan, which puts severe constraints on the size of signals that can be observed and presently limits its usefulness to the recording of relatively large signals from well established [Ca^2+^] indicator dyes.

## Discussion

Seventeen years after its introduction [1] two-photon imaging [21] is still too costly for most laboratories. This stems partly from the high cost of femtosecond lasers suitable for daily use, and partly from the rooting of the technique in traditional “eyepiece” microscopy—multiphoton excitation is still commonly viewed as an add-on to a confocal microscope, making integration with electrophysiological equipment difficult and severely limiting possible stimulation and recording protocols to the ones that fit within the traditional image-centric paradigm.

In this article we described a different approach, where the entire imaging system was designed synergistically from pump to photomultiplier, driven by the need to direct the probing beam to precisely defined locations in space at arbitrary times. This allowed the scarcest resource in functional imaging—the time available for photon collection—to be maximized. Benefiting from the convergence of several technologies, our design achieved a breadth of capabilities essential to an electrophysiologist at a fraction of the cost of less capable commercially packaged systems. Most importantly, its small size and operational simplicity make the system suitable for single-user mode of operation, which is better suited to electrophysiological inquiry than the currently prevailing “imaging facility” mode.

### Yb:KYW laser

Two recent advances that enabled the shrinking of our system were the development of lasers based on Ytterbium-doped double tungstates such as KY(WO_4_)_2_ (Yb:KYW) [22]–[26] and the invention of SEmiconductor Saturable Absorbing Mirrors (SESAM) [27], [28] for passive modelocking of laser cavities.

The high cross-section for stimulated emission of Yb:KYW makes it straightforward to construct a wide range of cavity configurations without Q-switching instabilities. Unlike Titanium, Ytterbium can be pumped by very quiet, efficient, reliable and inexpensive InGaAs/AlGaAs laser diodes used widely in telecommunications. The high thermal conductivity of the material and its low rate of heating per unit of output power allow the extraction of relatively high average powers without active cooling of the medium. In our system we used a single 3.5 W pump diode to get over 300 mW of very quiet single-mode femtosecond output—using only 8 W of electrical power! Diodes of much higher brightness are now commercially available, promising even higher output powers. Since the theoretical limits on extraction efficiency from Ytterbium-based materials can be over 90%, it is possible in principle to get over 10 W of average power from a laser as simple as ours [29]. With a reduced pulse repetition rate such a laser could reach imaging depths comparable to those achieved with a regenerative amplifier [30], perhaps even optical histology [31].

SESAM [27], [28] is a thin sliver of semiconductor, measuring a few millimeters on a side, glued onto a heatsink. When used in place of one of the mirrors inside a laser cavity it makes the cavity favor the propagation of femtosecond pulses over continuous light by absorbing photons less when their intensity is high. This standalone mirror does not connect to any external electronics and is therefore far simpler to deploy than the acousto-optic modulators still widely used in many broadly-tunable Ti:sapphire lasers; the pulsing can be self-starting and more stable than in other Ti:sapphire lasers modelocked through Kerr-lensing effects. Even more important for our design goals, the wide range of available SESAM parameters allowed us to choose from an equally wide range of pulse widths and repetition rates to fit within the limitations of the rest of the imaging system, which is difficult or impossible with standard Ti:sapphire lasers. We used a total of 0.8% of modulation depth, which resulted in >310 fs pulses. Much shorter pulses are achievable with greater modulation depth [22] with the theoretical limit of Ytterbium well under 20 fs and the practical record currently at 47 fs [32]. We were not able to achieve stable modelocking with a still lower modulation depth, indicating that it may be difficult to produce slightly longer pulses using this method. Much longer pulses could be produced by removing the prisms and operating the laser in picosecond mode.

Our laser resonator was similar to the ones commonly used in the construction of diode-pumped Ytterbium-based femtosecond lasers [22]–[26], [29] with one important difference: the modelocking device was placed in the same arm as the prisms that correct group velocity dispersion. This had several practical advantages for our application. It permitted the use of a longer-focus mirror M1 on the output side of the cavity (Figure 2a), and therefore a wider output beam, which eliminated the need for an external beam expander. At the same time the beam width and the divergence through the prisms could be kept low, allowing lower losses and a shorter inter-prism separation, therefore a shorter cavity with higher maximal pulse repetition rate. The mismatch between the focal lengths of mirrors M1 and M2 made the size and the location of the beam waist relatively insensitive to the length of the output arm, allowing us to change the pulse repetition rate over a wide range by simply moving the output coupler. Furthermore, two passes over the SESAM effectively doubled the modulation depth to a level sufficient for stable modelocking while the damage threshold stayed the same, which allowed the use of a SESAM with the highest damage threshold commercially available. Moreover, displacing the SESAM from the focus of the focusing cavity segment M3-M4 enables continuous adjustment of the incident power density, which makes it possible to increase pump power in the future without requiring a redesign of the rest of the cavity to avoid damage.

Lasers based on materials other than Ti:sapphire have been applied to two-photon imaging before: for example Cr:LiSAF at 860nm and Nd:YVO_4_ at 1064 nm [33], [34]. For various reasons these types of lasers failed to gain wider acceptance in biological imaging. In the case of Cr:LiSAF this was largely due to the material's limited maximal output power, and to competition with the already established Ti:sapphire product lines that covered the same spectral range. Neodymium-based lasers, on the other hand, lase at wavelengths too long to excite GFP, and their tuning range is narrow. In contrast, Yb:KYW permits the extraction of relatively high powers from simple cavities, and its gain bandwidth ranges from 920nm to almost 1100nm, a region where many fluorophores of interest to functional imagers become usable but where Ti:sapphire starts to require very powerful, inefficient and expensive pumping. Moreover, highly efficient [35] and inexpensive continuous-wave Ytterbium lasers can be used to pump Cr^4+^:forsterite and Cr^4+^:YAG, thus enabling access to the largely unexplored 1200−1500 nm wavelength range. Ytterbium therefore seems poised to end Titanium's decades-long dominance over biological multiphoton imaging.

### Acousto-optic scanning with Direct Digital Synthesis

Acousto-optic deflection (AOD) has many uses in optoelectronics and telecommunications, so compact and reliable deflectors are now commercially available in many configurations. The main impediment to their use in biological imaging with nonlinear excitation has been the spatial and temporal spread of femtosecond pulses upon passage through these highly dispersive media. This was exacerbated by the competition between laser manufacturers for shorter pulse durations, which led to the broad spectra of commonly used lasers, as dictated by the Heisenberg uncertainty principle. Various solutions to these problems have been found, notably the correction schemes for spatial dispersion using prisms [36], [37] and acousto-optic modulators [38], [39], and the precompensation of temporal spread with pre-chirping devices [36], [37], [39], [40]. One group [41] eschewed most corrections by restricting the available spectral bandwidth in a manually tunable Ti:sapphire laser. We minimized these problems by moving to longer wavelengths, where the temporal dispersion in TeO_2_ is nearly three times smaller than at 800 nm, and by designing the laser to produce pulses long enough to keep the resolution limit from spatial dispersion comparable to the time-bandwidth product of the deflector.

Another significant technological advance used in the design of our apparatus was the development of a highly versatile Direct Digital Synthesis (DDS) device. Unlike voltage-controlled oscillators traditionally used to drive AODs, the frequency accuracy of a DDS device is exceptional, as good as that of its clock source. In practice this translates into perfect repositioning accuracy of random access. The ability to perform digitally controlled frequency sweeps translates into fast full-frame scanning, as well as 3D random access capability. Further, DDS output can be digitally modulated in a variety of ways, or changed entirely through computer control in less than a microsecond. This may enable further improvement of deconvolution-based super-resolution techniques [42], [43] by allowing modulation of the point-spread function during imaging.

The transit time of the deflecting acoustic waves through the TeO_2_ crystals is on the order of ten microseconds, dictated by the size of the objective's back-aperture. This latency is the limiting factor on how fast the illumination could be switched on and off, as the DDS chip operates at timescales an order of magnitude faster. No light reaches the sample unless acoustic energy is being generated, so photodamage does not occur except during acquisition. Unlike mechanical scanners, this method can eliminate the need for shutters, “fly-back blanking” or similar methods for keeping high-power excitation light from reaching the tissue. Moreover, the diffraction efficiency of acousto-optic deflectors depends monotonically on the RF power injected, so DDS control allows a precise control the amount of power delivered to every scanned pixel.

### Three-dimensional scanning

Frequency-modulation of the acoustic waves injected into the deflectors results in cylindrical lensing, which translates into axial displacement of the focal spot, as well as a smooth lateral displacement of the focus over time. These two phenomena cannot be separated. The latter effect has been used for scanning lines much faster than possible with a sequential pixel-by-pixel scan [20]; we used it to achieve fast line-based full-frame scanning. The lensing effect has recently been used to implement a three-dimensional scanning system using four acousto-optic deflectors [44]. Our method is much simpler to implement in comparison, and requires a laser ten to fifty times less powerful to achieve the same fluorescence intensity with two-photon excitation.

It is important to note several idiosyncrasies of three-dimensional scanning when using our method, since these limit the possible scanning patterns. First, unlike random access in the nominal focal plane, where a constant-frequency wave is injected into the deflector [41], a frequency-modulated pattern is not static but rather moves at the speed of sound *v*, resulting in constant motion of the focal spot in the *x-y* plane. Therefore, if single-voxel 3D random access is desired, the scan must be cycled by repeatedly injecting the same frequency-modulated pattern at the interval equal to the objective back aperture size divided by the speed of sound *v*. Second, as the amount of excursion from the nominal focal plane is increased the lateral field of view shrinks, down to a point [44] at the extremes where the frequency is swept across the entire bandwidth of the deflector in the time it takes to fill the back aperture of the objective. Third, the converging or diverging beams suffer a small additional spherical aberration upon passage through infinity-corrected objectives.

### Scan control and data acquisition with an oscilloscope

Modern oscilloscopes have enough computing power to eliminate the need for a dedicated microscope controller and data acquisition system. Since they are aimed at a large market, they are a far less expensive and more versatile solution to novel imaging strategies than the design of a dedicated microscope controller.

In contrast to the requirements of digitizing electrophysiological measurements, where slower sampling at resolutions of more than eight bits is desirable, the physics of imaging fast processes typically constrains the amount of information in any given sample to far fewer than the eight bits available. Greater bit depths for sensitive measurements are easily achieved through shrewd use of the very high temporal resolution and the long sample memories now available. For example, by counting photons using photomultiplier tubes or avalanche photodiodes the ultimate bit-depth of optical measurements is limited only by the amount of time available for signal integration. With scanned illumination, therefore, the rate-limiting step is the shot noise or the photodamage, not the resolution of the data acquisition system. Fluorescence lifetime imaging provides a good example of how the superior temporal resolution of an oscilloscope can be used to render sample resolution irrelevant: the lifetime measurement takes only a single bit from each sample to infer photon arrival times and form the fluorescence decay histogram.

Fluorescence lifetime imaging is a powerful emerging technique that eliminates many systematic uncertainties inherent in intensity or color measurement (light source noise, bleaching, blinking, vibration, wavelength-dependent tissue absorption, etc.), and is particularly useful in resolving fluorophores with overlapping spectra, for instance in FRET measurements [5]. The method we use for fluorescence lifetime imaging is similar to Time-Correlated Single-Photon Counting (TPSPC) [45] but it is performed without any additional hardware. The advantages of our scheme over a dedicated TPSPC card are much lower cost, no need for system integration, and no dead time for photon detection. Moreover, since the time correlation measurement is done in software our technique can in principle be used with any photomultiplier no matter what its response function, as long as some information about photon arrival times can be extracted from the response.

### Future improvements

The design of our imaging apparatus can be adapted in many ways to the diverse needs of particular experimental circumstances. Laser power can be increased [29] to speed up access deeper into scattering tissue. Wavelength, repetition rate and pulse width can be adapted to a wider variety of functionally active fluorophores, as well as to a different type of deflector. The beam can be made wider to improve axial resolution with objectives of high numerical aperture, at the expense of increased shot noise of fast functional measurements resulting from the smaller excited volume.

A larger AOD can be used to get more resolvable points, and therefore a larger diffraction-limited field of view, minding the increased repositioning time. An acousto-optic modulator [38] or, simpler, a custom-cut prism [36] can be inserted diagonally across the input of the AOD to correct for all or most of the spatial dispersion, thus permitting the use of shorter pulses with higher peak power for a given average laser power, minding the relatively higher photodamage [9], [10].

To further improve the signal-to-noise ratio when recording fast voltage transients it is essential to develop detectors that can sustain higher light intensities with low readout noise. Photomultipliers at high gains used for photon counting are approaching the physical limits of materials. Several ways around this limitation exist: dividing light among several detectors, signal amplification from lower gains with low-noise high-bandwidth solid-state amplifiers, or the use of fast CCD cameras as non-imaging photon counters. Of course, a far greater improvement could come from the invention of practical dyes or fluorescent proteins that give larger signals [18], [19], [46].

The bandwidth and the sampling rate of the oscilloscope we used are already well beyond the needs of biological imaging, so an increase in either would be unlikely to open access to more neuroscientific questions. Most physiological observation involves episodic recording, however, so longer sample memories would provide greater flexibility in designing stimulation protocols. When selecting an oscilloscope it is therefore prudent to select one with a lower analog bandwidth and the maximal available length of sample memory.

Finally, the active components of the acousto-optic scanning subsystem could in principle be radically miniaturized. Combined with novel objective designs [47], [48], [49] and the ability to mechanically decouple the scanner from the laser with a hollow-core optical fiber [50], this could pave the way for a head-mounted scanner enabling microsecond three-dimensional random access in awake, behaving subjects.

## Methods and Materials

All procedures were carried out in accordance with animal protocols approved by the Salk Institute. Brain slices were prepared from Long-Evans rats and Thy1.2-EYFP mice following standard procedures. Animals were anesthetized with isoflurane and decapitated. Brain was swiftly extracted and cut into 300–350 µm thick section in ice-cold sucrose-based solution (in mM: sucrose 204, KCl 2.5, NaH_2_PO_4_ 1.25, NaHCO_3_ 28, CaCl_2_ 0.5, MgCl_2_ 7, dextrose 7). Slices were transferred to standard ACSF (in mM: NaCl 130, KCl 2.5, NaH_2_PO_4_ 1.25, NaHCO_3_ 25, dextrose 10) and kept at 34°C until use.

Neurons were patch-clamped using an Axon 700B amplifier and pClamp software. Electrodes of 2–4 MΩ resistance were filled with a K-methylsulfate-based intracellular solution (in mM: KMeSO_4_ 133, NaCl 4, KHCO_3_, ATP-Mg 4, GTP-Na 0.38). Water-soluble voltage-sensitive dye di-2-ANEPEQ [14] (Molecular Probes) was added to the intracellular solution in concentrations ranging from 5–100 µM. The tip of the pipette was filled with dye-free solution to minimize extracellular staining during approach [16].

The optical signal in Figure 5 was recorded through a 580 nm long-pass filter. No filters were used in Figure 6.
